# To drink or not to drink: A study of the association between rates of non‐drinkers and per drinker mean alcohol consumption in the Swedish general population

**DOI:** 10.1111/dar.13501

**Published:** 2022-06-07

**Authors:** Jonas Raninen, Michael Livingston, Jonas Landberg, Mats Ramstedt

**Affiliations:** ^1^ Swedish Council for Information on Alcohol and Other Drugs Stockholm Sweden; ^2^ Department of Clinical Neuroscience Karolinska Institutet Stockholm Sweden; ^3^ Centre for Alcohol Policy Research La Trobe University Melbourne Australia; ^4^ School of Social Sciences Södertörn University, Unit of Social Work Huddinge Sweden; ^5^ National Drug Research Institute Curtin University Melbourne Australia; ^6^ Department of Public Health Services Stockholm University Stockholm Sweden

**Keywords:** alcohol, collectivity, drinker, non‐drinker, survey

## Abstract

**Introduction:**

Understanding how the mean consumption per drinker and rates of non‐drinking interplay to form overall per capita alcohol consumption is imperative for our understanding of population drinking. The aim of the present study is to examine the association between rates of non‐drinkers and per drinker mean alcohol consumption in the Swedish adult population and for different percentiles of drinkers.

**Methods:**

Data came from a monthly telephone survey of drinking habits in the Swedish adult population between 2002 and 2013. Alcohol consumption and non‐drinking during the last 30 days were measured by beverage‐specific quantity‐frequency questions. Regression models estimated the association between the rate of non‐drinkers and per drinker volume on annual data. Auto‐regressive integrated moving average time‐series models estimated the association on monthly data.

**Results:**

A significant (*P* < 0.01) negative association (−0.849) was found between the rate of non‐drinkers and per drinker mean volume on annual data. A unit increase in non‐drinking was associated with a decline of 0.85 cl of pure alcohol among drinkers. This finding was mirrored across all percentiles of consumption. The semi‐log models found that a 1% unit increase in the rate of non‐drinkers was followed by a 2% reduction in per drinker mean consumption. Auto‐regressive integrated moving average time‐series models verified these results.

**Discussion and Conclusions:**

There is a significant association between the proportion of non‐drinkers and the amount of drinking among drinkers. The theory of collectivity of drinking cultures should also include the non‐drinking part of the population.

**Key point summary**

- There is a significant negative association between rates of recent non‐drinkers and how much alcohol drinkers consume.
- Changes in per capita alcohol consumption will render changes in both rates of recent non‐drinkers and per drinker consumption.
- This implies that there is a connection between the two groups leading to a synchronised behaviour.
- Successful promotion of shorter terms of non‐drinking that leads to an increased rate of non‐drinkers will also lead to reduced alcohol consumption among drinkers.

## 1 INTRODUCTION

In recent years, increasing rates of non‐drinkers have been observed in many countries, especially among youth [1, 2, 3] but also in the adult population [4]. Most cross‐sectional differences in population drinking between countries and regions in the world also stem from differences in rates of non‐drinkers [5]. An increasing prevalence of non‐drinking in the population will inevitably push overall per capita figures down, but it is not clear if increasing abstention is associated with less drinking among those who consume alcohol. Shield *et al*. mapped various drinking measures, including per capita consumption and abstinence rates, across countries in different regions of the world [6]. Their analyses did not focus on the association between various measures, but from their results, we can see that there seems to be a negative association between rates of life‐time abstainers and total per capita volume of alcohol consumed for both men and women, while no pattern emerges for rates of former drinkers [6]. Meng *et al*. analysed British alcohol consumption and abstinence rates using age‐period‐cohort modelling and found that there was an association between cohorts in consumption and abstinence rates, such that in cohorts with lower consumption, there were higher rates of abstainers and in cohorts with higher consumption there were lower rates of abstainers [7]. Mäkelä and Härkönen showed that between two time points when per capita alcohol consumption decreased in Finland, abstinence increased while regular drinking decreased [8]. These findings suggest a link between abstention rates and consumption levels per drinker, but this has not been a key focus of the literature so far.

This study will explore if there is an association between the rate of non‐drinkers during the last 30 days and the volume consumed in the drinking part of the population in Sweden. As a point of departure, we use the theory of collectivity of drinking cultures [9] which has been influential during the last decades both in forming alcohol policies and in the way researchers try to predict and understand changes in alcohol consumption at a population level [10]. In short, the theory states that alcohol consumption is a social phenomenon and that social interaction causes collective changes in drinking. Individuals are not thought of as isolated units when it comes to consuming alcohol, instead, their behaviour is considered to be highly influenced by peers in their social network. Skog argued that this strong social component driving alcohol consumption means that the entire population's alcohol consumption will ‘move as one’ [9, 11]. Based on empirical findings supporting this idea, the policy message that has been drawn is that reductions in per capita consumption of alcohol will reduce alcohol‐related harm since also heavy drinkers drink less when overall consumption drops [12].

As argued by Rehm, a major limitation with the theory is the omission of the non‐drinking sector and the subsequent exclusion of a large part of the population from the analysis [13]. This limitation is critical if drinkers and non‐drinkers actually influence each other in a similar manner as drinkers influence other drinkers. Such mutual influence seems plausible—a high proportion of non‐drinkers might mean fewer drinking occasions in a given social network, while fewer abstainers might encourage more drinking. There is however a shortage of studies that have addressed this question empirically and it has recently been argued that improving our understanding of this is imperative for our understanding of the link between per capita consumption and rates of harm [14]. On the basis of a longitudinal study, Rosenquist *et al*. presented some empirical evidence of a social influence on non‐drinking, at least in terms of increases in the likelihood of becoming a non‐drinker in social networks including many abstainers [15]. Furthermore, a study of alcohol consumption among Swedish adolescents suggested that although the impact of increasing abstention rates on the mean consumption among adolescents was marginal, their indirect impact on the observed trends, through social interaction, might be far greater [3].

Two main hypotheses are plausible here:

1. There is no association between rates of non‐drinkers and the per drinker mean consumption. On the basis of 12 European surveys on alcohol consumption, Hupkens *et al*. [16] showed that there is no systematic variation in the abstinence rates and the average consumption between countries. Similarly, Rossow and Clausen concluded that across 15 African countries, the mean consumption among drinkers was unrelated to the prevalence of current drinkers [17].
2. There is a negative association such that when rates of non‐drinkers increase the mean per drinker volume will decrease and vice versa. There seems to be association between rates of non‐drinkers and volume of consumption across cohorts [7] and regions in the world [6] and it is therefore plausible that there will be an association within a population over time. It is likely that this would work through the same social interaction mechanism outlined by Skog for changes in drinking [9], so that when more people do not drink this influences the consumption of people in their network to drink less and during periods of lower consumption the pressure to drink will also be lower which might render an increase in the prevalence of non‐drinkers.

Thus, the major aim of the present study is to examine the association between rates of non‐drinkers and per drinker mean consumption in the Swedish general adult population.

A further aim is to examine if an association exists across different levels of consumption, that is, if there is an association between rates of non‐drinkers and drinking levels among light, medium and heavy drinkers. The marked changes in overall mean consumption and rates of non‐drinkers in Sweden during the period 2002–2013 provides us with an excellent opportunity to test these hypotheses.

## 2 METHODS

### 2.1 Data

The data for this study comes from a telephone survey with questions about self‐reported drinking habits that were collected between 2002 and 2013. A nationally representative sample of the Swedish general population aged 16–80 years was randomly drawn every month. An organisation specialised in performing telephone surveys performed the interviews and sampling. Interviews were then conducted until 1500 respondents were interviewed each month, resulting in a repeated cross‐sectional sample of 18,000 respondents each year [18]. While this survey is ongoing, substantial changes in survey items and sampling methods in 2014 mean that more recent data are not fully comparable to the 2002–2013 waves, so our analyses focus on this early period. Some cases were excluded from the analyses due to missing values on the consumption questions resulting in a final sample of 211,030 (see Table 1 for a total number of respondents each year). Up to 30 contact attempts were made before the case was recorded as a non‐response. The monthly non‐response rates ranged between 40% and 60% during the study period, increasing during the last couple of years. The coverage rate of consumption when compared to sales data have, however, remained stable at around 40% [18].

**TABLE 1:** Number of respondents, percent non‐drinkers, mean per drinker alcohol consumption in centilitres 100% alcohol per month and consumption in different consumption segments for each year

Year (*n*)	% Non‐drinkers	Per drinker mean consumption	p25	p50	p75	p90	p95
2002 (*n* = 18,042)	22.6	53.4	11.6	28.9	61.6	115.8	171.9
2003 (*n* = 18,044)	23.4	48.8	11.6	28.1	59.3	107.2	156.3
2004 (*n* = 13,512)	23.7	48.7	11.0	27.5	58.0	107.6	156.8
2005 (*n* = 18,050)	24.0	47.2	11.1	26.1	57.6	104.7	148.0
2006 (*n* = 18,019)	23.9	47.6	10.5	25.8	57.6	106.4	156.0
2007 (*n* = 17,999)	24.4	46.6	10.5	26.2	56.8	103.3	146.0
2008 (*n* = 17,999)	24.9	47.4	9.7	24.9	55.1	103.9	152.6
2009 (*n* = 18,002)	25.2	46.4	9.8	23.8	53.9	101.0	147.6
2010 (*n* = 18,001)	27.2	45.0	9.9	23.9	52.3	98.0	140.9
2011 (*n* = 18,014)	29.0	46.1	9.9	23.5	53.8	100.7	149.3
2012 (*n* = 17,947)	26.1	46.6	9.9	24.0	52.3	96.9	148.2
2013 (*n* = 17,401)	25.0	47.0	9.6	23.9	52.8	99.6	151.5

### 2.2 Per drinker mean consumption

The consumption estimate was calculated for the sub‐sample of current drinkers (*n* = 157,274) excluding non‐drinkers from the analytical sample. This measure was derived from a beverage‐specific quantity and frequency scale applied to drinking during the last 30 days, combining questions on how often spirits, wine, beer and cider have been consumed during the last 30 days and the typical amount consumed in one occasion. The frequency questions were formulated in the same way for all types of beverages, as follows: *How often have you consumed beer/cider/wine/fortified wine/spirits during the last 30 days?* The response categories spanned ‘more or less every day’, ‘4–5 times a week’, ‘2–3 times a week’, ‘once a week’, ‘about 2–3 times’, ‘about once’ and ‘never’. The quantity questions response alternatives were specific for each beverage and customised to the different standard containers in which the beverages are sold in. The answers were then summarised into a measure of overall drinking during the last 30 days. To obtain a measure of pure alcohol, this measure was multiplied by the average alcohol strength of the beverages using information derived from sales data provided by the Swedish alcohol monopoly (for a more detailed description of the methods in Swedish see Ramstedt *et al*. [18]). Neither the quantity or frequency questions were altered between 2002 and 2013. As the reference period is only 30 days, distorting memory effects are assumed to be relatively small, at least in comparison with scales using 6 or 12 months as reference periods [19]. Possible effects from the use of self‐reported information, for example, underreporting of consumption, should also be the same for each year because the questions were not altered during the study period.

### 2.3 The different levels of drinking

The value for the 25th, 50th, 75th, 90th and 95th percentile for drinkers only was calculated and extracted for each year to examine the association between the rate of non‐drinkers and drinking at different consumption segments in the population.

### 2.4 Rate of non‐drinking

Current non‐drinkers were defined as those answering ‘never’ on all the beverage‐specific frequency questions described above, that is, they had not had a drink of alcohol during the 30 days prior to being surveyed. It is worth noting that past‐30‐day abstention is the only non‐drinking measure available in the monitoring survey, so we are limited to this measure for our estimated non‐drinking rates. This measure was calculated using the entire analytical sample (*n* = 211,030).

### 2.5 Analysis

We regressed the annual rate of non‐drinkers on the mean per drinker alcohol consumption for each year (*n* = 12) to examine if there was any association between the two measures. We then continued the analysis by doing the same for each of the different levels of consumption (the consumption value for the 25th, 50th, 75th, 90th and 95th percentile). These analyses were carried out both on the raw measures (linear models) and the logarithm of the outcome measure (semi‐log model). By doing so we obtained an estimate of both the absolute and the relative association.

As a further test of the association, we fitted auto‐regressive integrated moving average (ARIMA) time‐series models to monthly data (*n* = 132) of mean per drinker volume as the dependent variable and monthly rates of non‐drinkers as the independent variable. By increasing the number of observations (*n* = 132) these models increased the power of the analysis and by controlling for trends and seasonality in the data, the risk of obtaining spurious associations was substantially reduced [20].

The analysis was not pre‐registered and the results should therefore be considered exploratory.

## 3 RESULTS

**FIGURE 1:**
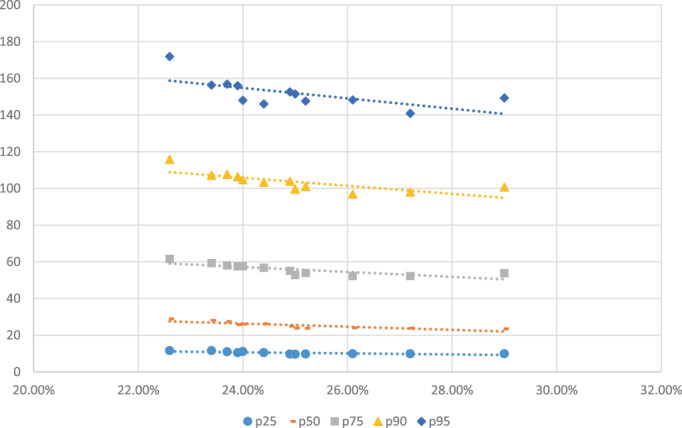
Average consumption at different percentiles each year sorted after the rate of non‐drinkers

Between 2002 and 2011, there was a steady increase in the rate of non‐drinkers from 22.6% to 29%. During the same time period, the per drinker mean consumption dropped by roughly 7 cl (100% ethanol). For the following 2 years, there was a decrease in the rate of non‐drinkers and a slight increase in mean per drinker consumption (see Table 1). Roughly, the same pattern was found for the mean consumption at different consumption levels (see Table 1 and Figure 1). The patterns emerging thus suggest that there is a negative association between the rate of non‐drinkers and the amount of drinking among those who consume alcohol.

These results were confirmed by estimations of linear regression models on annual data presented in Table 2; a significant (*P* < 0.01) negative association (−0.85) was found between the rate of non‐drinkers and the mean level of consumption among drinkers. This suggests that a 1% unit increase in non‐drinking was associated with an average decline in drinking of 0.85 cl of pure alcohol among drinkers. This result was echoed across different levels of drinking. The strongest association was found for the heaviest drinkers with a continuous decreasing estimate from high to low consumers. The reversed pattern was found in semi‐log models estimating the relative change in mean consumption where the lowest estimate was found for the heaviest drinkers. The results indicate that a 1% unit change in the rate of non‐drinkers on average corresponds to a 1.75% decline in consumption among drinkers.

**TABLE 2:** Results from linear regressions of rate of non‐drinkers on per drinker mean consumption and consumption at different percentiles, absolute and relative effect

	Coefficient	*P* > t	95% CI
*Absolute*			
Mean	−0.849	0.010	−1.448 to −0.249
p95	−2.870	0.023	−5.246 to −0.494
p90	−2.215	0.004	−3.564 to −0.867
p75	−1.351	0.002	−2.082 to −0.62
p50	−0.868	0.001	−1.295 to −0.441
p25	−0.291	0.011	−0.499 to −0.084
*Logarithmic*			
Mean	−1.750	0.009	−2.947 to −0.553
p95	−1.849	0.022	−3.364 to −0.335
p90	−2.11	0.004	−3.378 to −0.843
p75	−2.401	0.002	−3.698 to −1.104
p50	−3.372	0.001	−4.991 to −1.754
p25	−2.745	0.011	−4.714 to −0.776

CI, confidence interval.

As a complementary analysis, the association was also estimated on monthly data (*n* = 132) with ARIMA time‐series analysis. Two models using a specification that controls for trends and seasonality in the data were estimated—one linear and one semi‐log model (Table 3). The findings from these analyses are consistent with the results presented above based on a smaller number of annual observations and the confidence intervals of the estimates overlap in both cases. Both models were satisfactory with respect to model fit and have appropriate results in the test for uncorrelated residuals.

**TABLE 3:** Estimated association between non‐drinking (%) and alcohol consumption per drinker (litres 100%)

Model	Estimate	95% CIs	*p*	Q^a^	*p*(Q)	Model
Linear	−1.05	−1.55–0.56	<0.01	4.46	0.9	(1,0,0) (1,0,0,12)
Semi‐log	−2.12	−3.11–1.24	<0.01	4.26	0.9	(1,0,0) (1,0,0,12)

*Note*: Monthly data for 2002 to 2012 covering ‘the last 30 days’ (*n* = 132).

CI, confidence interval.

^a^ Q = Box‐Pierce test of residuals at lag 10.

## 4 DISCUSSION

The results of this study demonstrated a significant association between non‐drinkers and drinkers in the Swedish general population. The findings are supported both by regression analyses on yearly data and from the ARIMA time‐series analysis on monthly data. The results suggest that changes in one group are paralleled by changes in the other group so that increases in the number of non‐drinkers in the population are associated with less drinking among those who drink and vice versa. This suggests that the changes in overall per capita consumption observed in Sweden during the period 2002–2013 were the result of both changed drinking among those who drank and of changes in the proportion of non‐drinkers in the population, rather than solely being a result of variations in the proportion of non‐drinkers. A major conclusion is thus that the non‐drinking population and the drinking population should not be seen as two isolated phenomena but rather as one group with a collective behaviour that has a mutual influence on each other and change in concert. Previous studies have shown an increased likelihood of becoming a non‐drinker in networks with more abstainers [15], indicating a social contagion of the behaviour of not drinking. Our results extend this previous finding and indicate an interaction also impacting the behaviour of those still drinking alcohol.

A limitation of the analysis is the nature of the data that does not allow us to draw any conclusions about causality and thus we cannot say what precedes the other, that is, does a change in rates of non‐drinking give rise to a decline in consumption among drinkers or vice versa. In fact, both causal pathways are possible, that is, more non‐drinkers influence drinkers to consume less alcohol and less drinking among drinkers makes it easier to abstain from drinking alcohol. In Sweden, alcohol consumption is very associated with leisure time. During the holiday season over the summer months (June, July and August), consumption increases by an average of roughly 30% each year [21]. During this period, infrequent drinkers who normally do not drink are likely exposed to more drinking occasions and more occasions when it is culturally appropriate to drink and thus, they may be influenced to participate in one of their infrequent drinking occasions. Reversely, during a period like Sober October or White January, it is likely that people who are otherwise drinkers, abstain from alcohol. Both of these are examples of how social contagion could work to produce the empirical patterns that we observe in the present study, and of how these mechanisms could work in both directions.

It is also plausible that the observed association is the result of common exogenous factors affecting both non‐drinking and consumption levels among drinkers, that is, changes in income, price, availability or common changes in norms towards alcohol. Time‐series provides some protection against this since we also model the association with a seasonally adjusted model. Possible confounding factors therefore needs to be associated with the two variables in a consistent pattern over time rather than merely impacting the levels or trends in the two measures.

It should also be noted that the results demonstrated a close temporal pattern between the series so that if an exogenous factor pushes the rate of non‐drinking to increase, this may influence the current drinkers to reduce their consumption at the same time. So, regardless of the precise causal pathway, the findings suggest that the two groups are closely associated and should be looked upon as one group with a collective behaviour.

In Skog's theory, it was the behaviour of drinking that was social and therefore collective. In his work, Skog excluded the non‐drinkers as the behaviour under study was the consumption of alcohol. Skog also identified what he called ‘barriers of diffusion’; borders over which the transmission of the behaviour (drinking) had problems crossing [11]. One obvious barrier is the border between drinkers and non‐drinkers and therefore our results might seem somewhat surprising. Historically in the Nordic countries, not drinking has also been a non‐normative behaviour and seen as something socially deviant [22, 23]. The increase in abstinence rates during the last decades has however introduced new groups of abstainers [24] and it is plausible that non‐drinking has then become a less deviant behaviour today and that this group has become a more integrated part of the population [25]. This could then lead to a reduction in the barriers of diffusion and increase the possibility of mutual influence of the groups on each other. The sharp increases in non‐drinking observed among young people over the last couple of decades [1, 26] might also have implications for the interpretation and implications of our findings, as these historically dry cohorts are now ageing into adulthood. Cohort effects have previously been observed for drinking behaviours [27, 28, 29] so it is reasonable to assume that these dry cohorts, with high rates of non‐drinkers, can impact the total per capita consumption and also impact the drinking part of the population.

The results that the largest effect in absolute changes is found for heavy drinkers while the opposite is true for relative changes are in line with what was found in a study of changes in drinking among Swedish youth [3]. A small relative change for the heaviest drinking group should be mirrored by a large absolute change simply because of the amount drunk by this group. It is however somewhat surprising that the strongest association with the rate of non‐drinkers was found for the heaviest drinking group since one would expect the social interaction to be the weakest between this group and the group of non‐drinkers. One possible explanation is that this is a statistical artefact; the changes in drinking are collective from light to heavy drinkers and thus we should expect the same association with rate of non‐drinkers for both light and heavy drinkers, but since consumption is higher among heavy drinkers, the coefficient for this groups becomes inflated and produces a stronger association. Even if this is the case the results imply that there is an association between abstainers and heavy drinkers and that through the proposed social interaction, heavy drinkers are influenced to drink less when the rate of non‐drinkers in a population increases, and the rate of non‐drinkers will decline when consumption among the heavy drinkers increase.

The findings indicate that a 5% increase in non‐drinkers would be mirrored by an approximate 9% reduction in drinking among drinkers. For Sweden in 2013, this corresponded to a reduction of 0.89 L of pure alcohol per capita. Given the association between volume consumed per capita and alcohol‐related harm [30, 31, 32], this in turn could potentially render positive public health gains. In fact, the findings suggest that prevention efforts aiming at increasing non‐drinking in the population may be worth considering more in the future.

The short reference period of the last 30 days used in the study does not allow us to separate out life‐time abstainers from others that usually or occasionally consume alcohol but had not during the last month prior to being interviewed. This means that the group ‘non‐drinkers’ will constitute a rather heterogeneous group. On the other hand, our focus was to assess the link between non‐drinking and drinking during a specific time‐period and whether non‐drinkers are life‐time abstinent or not is not crucial in this context. From a public health and policy perspective, this is also interesting since even the successful promotion of shorter terms of non‐drinking could have positive effects on a population level, rather than trying to promote long‐term abstinence from alcohol. Future studies should however examine if prevalence of different types of non‐drinkers differ in their association with the per drinker mean consumption.

It should be kept in mind that the results are derived from self‐reported information which usually includes underreporting, because of for example socially desirable answers and recall bias [33, 34]. Another problem with surveys is sampling bias and bias stemming from response rate. The response rate of the survey was declining during the studied period, however, the coverage rate when compared to registered sales data has been fairly stable at approximately 40% [18] and since the mode of collection and sampling has not been altered during the study period these problems and their possible impact on the results should be relatively constant. With our focus being on changes and association over time these problems should be less salient [35].

One of the biggest strengths of the study is the large sample at hand and that data has been continuously collected in a consistent manner. This allowed us to test the association between rates of non‐drinkers and the mean consumption among drinkers on temporal data, it also enabled us to elucidate if there was an association in the changes of the two phenomena. This however limited the study period to the years 2002–2013 since there have been changes in how data is collected after that making the more recent years not fully comparable. This means that possible effects introduced by the COVID‐19 pandemic, for example, on the association between non‐drinkers and drinkers are overlooked. In general, we would argue that the association under study here is not time‐dependent, making up‐to‐date data less salient for our research purposes. We do however think that the COVID‐19 pandemic might be an exception since it has so fundamentally altered how we socialise and in what contexts and social spheres alcohol is consumed. Future studies with access to pre‐, during‐ and post‐pandemic data will be able to study this in detail. Something that further strengthens the findings is that all analyses of both annual and monthly data corroborated the findings.

Our results have shown a significant association between the rate of non‐drinkers during the last 30 days and the mean volume of alcohol consumption in the drinking part of the population in Sweden. Further studies looking at this association cross‐culturally are warranted to corroborate these findings outside of Sweden. Across 15 African countries, there was no systematic variation in the mean consumption among drinkers and the prevalence of current drinkers [17], indicating that the associations observed in the present paper are not valid in this context with higher rates of non‐drinking and where alcohol consumption is a less normative behaviour. How these findings can be incorporated into the theory of collectivity of drinking cultures should be examined.

Skog's theory has been the subject of much scholarly debate over the years with critics arguing that the theory is too vague to allow for empirical tests and that the shape of the distribution of consumption is different from that suggested by Skog [13, 36, 37, 38, 39]. Others have also pointed out that one central aspect missing from the theory is that it does not incorporate non‐drinkers [10, 13]. Our results indicate that the most important question of distinction is not whether ‘to drink or not to drink’, since drinkers and non‐drinkers move in concert when drinking change. Therefore, they do not seem to be two separate populations co‐existing isolated from each other and they can instead be looked upon as one group with a collective behaviour.

## 5 CONCLUSION

There is a significant and negative association between rates of recent non‐drinkers and per drinker mean alcohol consumption in the Swedish adult population. When per capita alcohol consumption increases, both the rate of recent non‐drinkers declines and the per drinker mean consumption increase. Conversely, when per capita consumption decreases, both the rate of recent non‐drinkers increase and the per drinker mean consumption decline. These results were found in both annual and monthly data.

## AUTHOR CONTRIBUTIONS

Jonas Raninen conceived and planned the study in collaboration with Michael Livingston. Jonas Raninen put together the database, the descriptive analyses and performed the regression analyses. Mats Ramstedt performed the time‐series analyses. Jonas Raninen wrote the first draft, Mats Ramstedt, Michael Livingston and Jonas Landberg commented and made revisions and then Jonas Raninen wrote the final draft. All authors have read and approved the final version.

## CONFLICT OF INTEREST

We declare that none of the authors are in receipt of financial support or has any relationship that may pose a conflict of interest in relation to the content presented in the submitted manuscript.
